# Clinical value of plasma pTau181 to predict Alzheimer's disease pathology in a large real-world cohort of a memory clinic

**DOI:** 10.1016/j.ebiom.2024.105345

**Published:** 2024-09-18

**Authors:** Amanda Cano, María Capdevila, Raquel Puerta, Javier Arranz, Laura Montrreal, Itziar de Rojas, Pablo García-González, Claudia Olivé, Fernando García-Gutiérrez, Oscar Sotolongo-Grau, Adelina Orellana, Nuria Aguilera, Maribel Ramis, Maitee Rosende-Roca, Alberto Lleó, Juan Fortea, Juan Pablo Tartari, Asunción Lafuente, Liliana Vargas, Alba Pérez-Cordón, Nathalia Muñoz, Ángela Sanabria, Montserrat Alegret, Xavier Morató, Lluís Tárraga, Victoria Fernández, Marta Marquié, Sergi Valero, Daniel Alcolea, Mercè Boada, Agustín Ruiz

**Affiliations:** a Research Center and Memory Clinic, Ace Alzheimer Center Barcelona, Barcelona, Spain; b Biomedical Research Networking Centre in Neurodegenerative Diseases (CIBERNED), Madrid, Spain; c Department of Pharmacology, Toxicology and Therapeutic Chemistry, Faculty of Pharmacy and Food Science, Universitat de Barcelona, Spain; d Sant Pau Memory Unit, Department of Neurology, Hospital de la Santa Creu i Sant Pau, Biomedical Research Institute Sant Pau (IIB Sant Pau), Universitat Autònoma de Barcelona, Barcelona, Spain; e Barcelona Down Medical Center, Fundació Catalana Síndrome de Down, Barcelona, Spain; f Glenn Biggs Institute for Alzheimer's & Neurodegenerative Diseases, University of Texas Health Science Center, San Antonio, TX, USA

**Keywords:** Plasma biomarkers, pTau181, Alzheimer's disease, Prodromal AD, Mild cognitive impairment, Real-world cohort

## Abstract

**Background:**

The identification of patients with an elevated risk of developing Alzheimer's disease (AD) dementia and eligible for the disease-modifying treatments (DMTs) in the earliest stages is one of the greatest challenges in the clinical practice. Plasma biomarkers has the potential to predict these issues, but further research is still needed to translate them to clinical practice. Here we evaluated the clinical applicability of plasma pTau181 as a predictive marker of AD pathology in a large real-world cohort of a memory clinic.

**Methods:**

Three independent cohorts (modelling [n = 991, 59.7% female], testing [n = 642, 56.2% female] and validation [n = 441, 55.1% female]) of real-world patients with subjective cognitive decline (SCD), mild cognitive impairment (MCI), AD dementia, and other dementias were included. Paired cerebrospinal fluid (CSF) and plasma samples were used to measure AT(N) CSF biomarkers and plasma pTau181.

**Findings:**

CSF and plasma pTau181 showed correlation in all phenotypes except in SCD and other dementias. Age significantly influenced the biomarker's performance. The general Aβ(+) *vs* Aβ(−) ROC curve showed an AUC = 0.77 [0.74–0.80], whereas the specific ROC curve of MCI due to AD *vs* non-AD MCI showed an AUC = 0.89 [0.85–0.93]. A cut-off value of 1.30 pg/ml of plasma pTau181 exhibited a sensitivity of 93.57% [88.72–96.52], specificity of 72.38% [62.51–79.01], VPP of 77.85% [70.61–83.54], and 8.30% false negatives in the subjects with MCI of the testing cohort. The HR of cox regression showed that patients with MCI up to this cut-off value exhibited a HR = 1.84 [1.05–3.22] higher risk to convert to AD dementia than patients with MCI below the cut-off value.

**Interpretation:**

Plasma pTau181 has the potential to be used in the memory clinics as a screening biomarker of AD pathology in subjects with MCI, presenting a valuable prognostic utility in predicting the MCI conversion to AD dementia. In the context of a real-world population, a confirmatory test employing gold-standard procedures is still advisable.

**Funding:**

This study has been mainly funded by Ace Alzheimer Center Barcelona, 10.13039/501100004587Instituto de Salud Carlos III (ISCIII), Biomedical Research Networking Centre in Neurodegenerative Diseases (CIBERNED), Spanish Ministry of Science and Innovation, Fundación ADEY, Fundación Echevarne and Grífols S.A.

**Research in context**

**Evidence before this study**

We searched PubMed for all articles published from database inception to November 1, 2023, with no language limitations. Keywords included: “plasma phosphorylated tau”, “blood phosphorylated tau” “tau-PET”, “CSF phosphorylated tau”, “Alzheimer's disease”, “Alzheimer's pathology”, “prodromal Alzheimer's disease”, “preclinical Alzheimer's disease”, “mild cognitive impairment”, and “clinical cohort”. CSF tau phosphorylated at threonine 181 (pTau181) is one of the core biomarkers incorporated into the National Institute on Aging—Alzheimer's Association Research Framework to define Alzheimer's disease. In plasma, most of the previous studies have been carried out with selected cohorts of HC, patients with MCI and AD dementia. In this sense, cross-sectional studies have highlighted the potential of plasma pTau181 to be used as a screening tool for patients with high-risk of AD. A good correlation of plasma pTau181 with its homolog in CSF have been already reported, as well as the increased levels of pTau181 levels when compared HC and subjects with AD dementia. Similar results were observed between subjects with MCI Aβ(+) and MCI (Aβ−). However, many studies were not able to determine a cut off value and provide information about the sensitivity and specificity of the biomarker, did not possess gold standard references (CSF or PET biomarkers) to test the findings, or did not included validation cohorts. Regarding the conversion to dementia, previous studies have reported that blood pTau181 had a high diagnostic accuracy for differentiating AD from non-neurodegenerative causes and predicted amyloid status. However, few of these studies were performed in real-world subjects of a memory clinic.

**Added value of this study**

The most important value of this study is the generation of robust clinical data of the plasma pTau181 behaviour in one of the largest and well characterised real-world cohort of patients worldwide, with more than 2000 paired CSF/plasma samples and clinical data. Furthermore, three independent cohorts of patients were included to modelled, tested and validated the obtained results. To the best of our knowledge, this is the largest study of plasma pTau181 in real-world patients, providing evidence of the clinical value of plasma pTau181, in the CoU of screening subjects with increased risk of AD pathology, in real-world patients diagnosed at a memory clinic. Our results demonstrated that plasma pTau181 significantly correlated with its CSF pTau181 as previously described, in all phenotypes but not in SCD and other dementias' subjects, which highlighted the AD specificity of pTau181 and suggested that another biomarker would be needed to address AD pathology in the healthy populations. Age, Aβ(+) status, ApoE ε4 and MMSE scores appeared to significantly be correlated with the biomarker's performance. A specific cut-off value for patients with MCI revealed that plasma pTau181 exhibited sensitivity of 93.57% [88.72–96.52], specificity of 72.38% [62.51–79.01], VPP of 77.85% [70.61–83.54], and 8.30% false negatives when discriminated between patients with MCI due to AD and non-due to AD. This accuracy would mean 38.83% fewer lumbar punctures for this phenotype, together with important saving costs for the memory clinic and health systems. Moreover, plasma pTau181 exhibited a great accuracy to predict MCI conversion to AD dementia. Subjects with MCI who converted to AD dementia showed significantly increased plasma pTau181 levels. In addition, those patients with plasma pTau181 levels above the cut off value exhibited a HR of 1.84 [1.05–3.22] higher risk of converting to AD dementia. In contrast, 80% of the patients with MCI and plasma pTau181 levels under the cut-off value remained stable during a 5-years follow up period.

**Implications of all the available evidence**

This study shows that the evaluation of plasma pTau181 (as well as others plasma biomarkers) in real-world cohort is crucial to understanding the relative performance and utility of plasma pTau181 biomarker in the clinical routine of the memory clinics. These data, together with other recent reports, suggest that pTau181 is a useful biomarker to identify AD pathology in subjects with MCI. Plasma biomarkers are likely to be less expensive and more accessible than CSF or PET biomarkers, allowing them to be more easily deployed and more scalable to the clinical practice. Being CSF pTau181 the gold standard biomarkers used from a clinical point of view, plasma pTau181 represents a particularly powerful tool for differential diagnosis between AD and other dementias in the MCI phase of the disease continuum. In turn, this could further improve the ability to identify suitable participants for clinical trials, DMTs and, definitely, achieve the current paradigm of precision medicine.

## Introduction

The economic and social costs of the dementia epidemic threaten the sustainability of healthcare systems around the world.^1^ Alzheimer's disease (AD), the main underlying cause of dementia in the elderly, is responsible for 60–80% of the total number of dementia cases. AD is the only condition among the 10 principal mortality causes worldwide that is still without a preventative treatment or cure.^1^

It is well described that the molecular alterations start in the preclinical stages, when the clinical symptoms are not appreciable. In the prodromal phase, also described as mild cognitive impairment (MCI) due to AD, first clinical symptoms appear and the levels of amyloid-β peptide (Aβ) and hyperphosphorylated tau (pTau)-mediated neuronal injury increase exponentially. Since neuronal damage is considered irreversible with currently available treatments, the identification of these patients in the earliest stages is one of the greatest challenges in clinical practice. With the recent arrival of the first disease-modifying treatments (DMTs), the identification of eligible patients will help to reduce the predicted worldwide socioeconomic tsunami of burdens associated with dementia.^2^

The invasive nature and high cost of current clinical AD biomarkers (positron emission tomography (PET) radiotracers and cerebrospinal fluid (CSF) biomarkers^3^) has promoted a growing scientific interest in plasma biomarkers.^4^ The Alzheimer's Association has recently highlighted the revolution of plasma biomarkers will mean for AD prognosis and diagnosis, as well as the improvement in the design of interventional trials.^5^ However, they also remarked that further research is still needed before plasma biomarkers can be translated to the memory clinics.^6^

Recently, several promising plasma biomarkers have been studied in a number clinical cohorts, such as plasma the Aβ42/40 ratio,^7^, ^8^, ^9^ NfL,^10^^,^^11^ GFAP,^12^, ^13^, ^14^ or different pTau isoforms, such as pTau181, pTau217, or pTau231.^15^, ^16^, ^17^ Various studies have shown that plasma pTau isoforms are highly accurate and specific for the detection of tau pathology and PET-confirmed amyloidosis across the clinical continuum of AD.^18^, ^19^, ^20^ However, the most widely studied CSF/PET reference of tau phosphorylation in the AT(N) classification is the pTau181 isoform.^6^ Many studies have already highlighted the relevance of plasma pTau181 in different CoUs, such as diagnosis, trial selection, and disease monitoring of patients with high-risk of AD.^21^, ^22^, ^23^ Furthermore, plasma pTau181 has also been reported to be increased in Aβ-PET positive but still tau-PET-negative subjects, which suggests a specific sensitivity of plasma pTau181 for early AD pathology.^19^

In this work, we present a cross-sectional study on three large independent cohorts with more than 2000 real-world patients diagnosed in a memory clinic. Having access to more than 1800 CSF/plasma paired samples obtained on the same day, we evaluated the clinical performance of plasma pTau181 in the CoU of the screening of AD pathology in subjects arriving routinely at the memory clinic, mostly from primary care centres or awareness campaigns.^24^^,^^25^ We aimed to evaluate whether plasma pTau181 can: (i) accurately detect AD pathology in subjects endorsed with different syndromic diagnosis (healthy with or without SCD, MCI, and overt dementia) and (ii) predict future cognitive decline and conversion to AD dementia in patients with MCI.

## Methods

### Standard protocol approvals, registrations and patient consents

All study protocols have been approved by the Clinical Research Ethics Commission of the Hospital Clinic (Barcelona, Spain, reference num: HCB/2014/0494) in accordance with the Declaration of Helsinki and the current Spanish regulations in the field of biomedical research (law 14/2007, royal decree 1716/2011). Likewise, in accordance with Spain's Data Protection Law (organic law 3/2018), all participants were informed about the study's goals and procedures by a neurologist before signing an informed consent form. Patients' privacy and data confidentiality were protected in accordance with applicable laws.

### Participants, study groups, and selection criteria

All samples available in our paired CSF/plasma collection (registered in the ISCIII with the code C.0000299, 2016–2022) were included. Participants were real-world patients evaluated and diagnosed at the memory clinic of the Ace Alzheimer Center Barcelona (ACE) (Barcelona, Spain), which belongs to the public health system of Catalonia region (Spain). Most patients were referred to ACE from primary care centres in the Barcelona metropolitan area. Detailed diagnostic criteria are described in the Supplementary Material. Briefly, a consensus diagnosis was assigned to each patient by a multidisciplinary team of neurologists, neuropsychologists, and social workers. Voluntary (with informed consent) lumbar punctures were offered to (i) individuals with MCI and dementia who were evaluated at the Memory Clinic of ACE^25^; (ii) participants of the Fundaciò ACE Healthy Brain Initiative (FACEHBI) with subjective cognitive decline (SCD); and (iii) participants of the BIOFACE study, which encompass individuals with early-onset MCI.^26^^,^^27^ All clinical diagnosis consisted of a primary, secondary, and a syndromic diagnosis, accompanied by a biological diagnosis with CSF biomarkers and the AT(N) classification.^25^^,^^28^

Three independent cohorts were defined in this study: i) modelling cohort (n = 991); ii) testing cohort (n = 642); and iii) validation cohort (n = 441), which was composed of the GR@CE/DEGESCO cohort^29^^,^^30^ (diagnosed at ACE as previously described) and the SPIN cohort,^31^ assessed at the Hospital Sant Pau Memory Unit (Barcelona, Spain). Sex data was collected and used as covariable in the study. Gender data (e.g. self-reported by study participants) was not collected nor taken into account during the design of the study. The demographic, clinical, neurological, and biological characteristics of the three independent cohorts are presented in Table 1. The study groups’ distribution is shown in Table 2. The percentage of the studied clinical variables is shown in the Table S1.

**Table 1:** Groups’ distributions of the study.

Distribution by phenotypes			
Study groups	ATN	Primary diagnosis	Syndromic diagnosis
SCD	–	SCD	SCD
MCI Aβ(+)	A + T (+/−)N (+/−)	MCI probable/possible	MCI
MCI Aβ(−)	A-(T+/−)N (+/−)	MCI probable/possible	MCI
AD dementia	A + T + N (+/−)	AD probable/possible	Dementia
Other dementias^a^	A-T-N (+/−)	–	Dementia

MCI distribution			
MCI sub-study groups	A	T	N
Negative profile	–	–	–
SNAP	–	+	+
SNAP	–	+	–
SNAP	–	–	+
Brain amyloidosis	+	–	–
Prodromal AD^b^	+	+	–
Prodromal AD	+	–	+
Prodromal AD	+	+	+

a Other dementias: frontotemporal dementia, Lewy body dementia, subcortical vascular disease dementia, psychiatric disease, cortico-basal degeneration, depression.

b Prodromal AD is referred to those subjects with MCI due to AD.

**Table 2:** Demographic, clinical, neurological, and biological characteristics of study cohorts.

Demographics	Modelling cohort			Testing cohort			Validation cohort					
	ACE CSF_1			ACE CSF_2			GR@CE/DEGESCO			SPIN		
	Mean (SD)	Median	IQR (Q1, Q3)	Mean (SD)	Median	IQR (Q1, Q3)	Mean (SD)	Median	IQR (Q1, Q3)	Mean (SD)	Median	IQR (Q1, Q3)
n	991			642			301			140		
Female (%)	59.7			56.2			55.1			57.0		
Age (years)	72.4 (9.4)	74.0	10.7 (68.0, 78.6)	71.9 (8.7)	72.8	12.3 (65.9, 78.2)	74.4 (8.9)	75.6	10.5 (69.7, 80.2)	71.4 (8.7)	73.5	10.90 (66.3, 77.3)
Education (years)	8.1 (4.7)	8.0	5 (6.0, 11.0)	8.6 (4.8)	8.0	5 (6.0, 11.0)	8.2 (4.1)	8.0	4.0 (6.0, 10.0)	11.9 (5.3)	11	8.0 (8.0, 16.0)
BMI (kg/m^2^)	26.4 (3.8)	26.4	4.7 (23.9, 28.6)	26.8 (3.8)	26.5	4.7 (24.2, 28.9)	27.0 (4.3)	26.8	5.2 (24.0, 29.3)	NA		
Race/ethnicity	NA			NA			NA			NA		

Syndromic diagnosis	Modelling cohort	Testing cohort	Validation cohort	
	ACE CSF_1	ACE CSF_2	GR@CE/DEGESCO	SPIN
	n (%)	n (%)	n (%)	n (%)
SCD	67 (6.8)	51 (8.0)	–	–
MCI total/MCI with follow up	606 (61.1)/388 (39.1)	433 (67.4)/374 (58.2)	301 (100.0)/225 (74.7)^a^	140 (100.0)/0 (0.0)
Dementia	318 (32.1)	158 (24.6)	–	–

CSF biomarkers (pg/ml)	Modelling cohort			Testing cohort			Validation cohort					
	ACE CSF_1			ACE CSF_2			GR@CE/DEGESCO			SPIN		
	Mean (SD)	Median	IQR (Q1, Q3)	Mean (SD)	Median	IQR (Q1, Q3)	Mean (SD)	Median	IQR (Q1, Q3)	Mean (SD)	Median	IQR (Q1, Q3)
Aβ_1-42_	812.2 (388.7)	695.5	480.8 (536, 1016.8)	842.2 (417.8)	719.5	543 (532, 1075)	917.5 (433.3)	781.0	628 (582.5, 1210.5)	792.4 (405.3)	678.5	495.0 (486.3, 981.3)
Aβ_1-40_	12410.1 (3978.3)	11768.5	5398.3 (9473.3, 14871.6)	12633.4 (4113.9)	12092.8	5181.1 (9687.2, 14868.3)	12,880 (3526.2)	12721.3	4559.3 (10415.3, 14974.6)	11313.0 (4218.7)	10500.5	5982.0 (8085.5, 14067.5)
pTau181	76.1 (50.7)	61.0	53.0 (42.0, 95.0)	69.9 (44.3)	57.0	51.0 (38.0, 89.0)	73.3 (44.5)	57.0	63.5 (39, 102.5)	73.2 (55.3)	52.5	49.3 (37.8 87.0)
tTau	487.7 (343.2)	384.0	344.0 (264.8, 608.8)	449.7 (290.8)	372.5	347.0 (238.0, 585.0)	461.6 (241.4)	380.0	309.5 (284.5, 594.0)	485.3 (312.8)	382.5	317.8 (274.0, 591.8)

Plasma biomarkers (pg/ml)	Modelling cohort			Testing cohort			Validation cohort					
	ACE CSF_1			ACE CSF_2			GR@CE/DEGESCO			SPIN		
	Mean (SD)	Median	IQR (Q1, Q3)	Mean (SD)	Median	IQR (Q1, Q3)	Mean (SD)	Median	IQR (Q1, Q3)	Mean (SD)	Median	IQR (Q1, Q3)
plasma pTau181	1.9 (0.8)	1.7	1.2 (1.2, 2.4)	1.8 (0.9)	1.5	1.2 (1.1, 2.3)	1.6 (0.7)	1.4	0.9 (1.0, 1.9)	2.5 (1.0)	2.3	1.3 (1.8, 3.1)

AT(N) classification	Modelling cohort	Testing cohort	Validation cohort	
	ACE CSF_1	ACE CSF_2	GR@CE/DEGESCO	SPIN
	n (%)	n (%)	n (%)	n (%)
A + T + N+	271 (27.3)	198 (30.8)	44 (14.6)	45 (32.0)
A + T + N-	108 (10.9)	51 (8.0)	10 (3.3)	6 (4.3)
A + T-N+	10 (1.0)	7 (1.1)	0 (0.0)	0 (0.0)
A + T-N-	145 (14.6)	82 (12.8)	10 (3.3)	17 (12.3)
A-T-N-	271 (27.4)	206 (32.0)	49 (16.3)	62 (44.0)
A-T-N+	15 (1.5)	11 (1.7)	3 (1.0)	4 (2.9)
A-T + N+	122 (12.3)	59 (9.2)	5 (1.7)	6 (4.3)
A-T + N-	47 (4.7)	25 (3.9)	0 (0.0)	0 (0.0)
NA	2 (0.2)	3 (0.5)	180 (59.8)	0 (0.0)

APOE	Modelling cohort	Testing cohort	Validation cohort	
	ACE CSF_1	ACE CSF_2	GR@CE/DEGESCO	SPIN
	n (%)	n (%)	n (%)	n (%)
ε2ε2	2 (0.2)	1 (0.2)	0 (0.0)	0 (0.0)
ε2ε3	49 (4.9)	38 (5.9)	19 (6.3)	10 (7.2)
ε2ε4	13 (1.3)	14 (2.2)	5 (1.7)	2 (1.4)
ε3ε3	437 (44.1)	325 (50.6)	129 (42.9)	96 (69.0)
ε3ε4	221 (22.3)	148 (23.1)	59 (19.6)	28 (20.3)
ε4ε4	27 (2.7)	31 (4.8)	2 (0.7)	3 (2.2)
NA	242 (24.3)	85 (13.2)	87 (28.9)	1 (0.7)

Clinical data	Modelling cohort			Testing cohort			Validation cohort					
	ACE CSF_1			ACE CSF_2			GR@CE/DEGESCO			SPIN		
	Mean (SD)	Median	IQR (Q1, Q3)	Mean (SD)	Median	IQR (Q1, Q3)	Mean (SD)	Median	IQR (Q1, Q3)	Mean (SD)	Median	IQR (Q1, Q3)
MMSE	24.4 (4.5)	26.0	7.0 (21.0, 28.0)	25 (4.1)	26.0	6.0 (22.0, 28.0)	26.3 (3)	27.0	3.8 (25, 28.8)	25.5 (3.2)	26.0	4.0 (24, 28)
MCI Conversion (%)	20.9			16.8			31.6			NA		

a From 225 of MCI with follow up data, 180 did not have AT(N) biomarkers.

### Plasma and CSF sample collection

Samples from Modelling, Testing and Validation-GR@CE/DEGESCO cohorts were measured at ACE research centre. Samples from Validation-SPIN cohort were measured at the Hospital San Pau Memory Unit.^32^ Plasma and CSF samples were collected on the same day from each patient as described elsewhere.^27^^,^^33^ Briefly, blood samples were collected in polypropylene tubes with EDTA (BD Vacutainer). Plasma was separated by centrifugation, aliquoted, and stored at −80 °C until use. CSF was obtained by lumbar puncture (LP) in polypropylene tubes (Sarstedt Ref 62.610.018), centrifuged for common AD biomarker determination, and the supernatant was aliquoted and stored at −80 °C until use. The collection protocol followed the recommendations of the Alzheimer's Biomarkers Standardization Initiative.^34^

### Analysis of biomarkers

In all cohorts, on the day of the analysis, a CSF/plasma aliquot was thawed and used for the determination. Quantification of Aβ_1-40_, Aβ_1-42_, t-Tau, and pTau181 CSF biomarkers were performed with the Lumipulse G 600 II automatic platform (Fujirebio Inc.) or a standard ELISA immunoassay (INNOTEST®, Fujirebio Europe, Göteborg, Sweden) as described elsewhere.^33^^,^^35^ Plasma pTau181 was measured with the Lumipulse G1200 automatic platform (Fujirebio Inc.) in the ACE and GR@CE/DEGESCO biospecimens, and Lumipulse G 600 II in the San Pau Memory Unit.^32^

### Statistical analysis

All statistical analyses were conducted using R studio version 4.0.3, SPSS, and GraphPad Prism 8.0. Risk stratification of the subjects from each study group were performed by the [A/T/(N)] classification as described elsewhere.^33^ Data were log-normalised and Z-score standardised, and the values of outliers were removed (+1.5IQR from Q3/−1.5IQR from Q1). Heterogeneity was evaluated with a sensitivity analysis, which showed no differences with the outliers' removal (Table S2). Proxy cut-offs were obtained by plotting receiver operating characteristic (ROC) curves (CSF/plasma biomarker level as predictor and conversion as outcome) and calculating the Youden index, which is the threshold value that provided the best trade-off between sensitivity and specificity, using the *roc* and *coords* functions from the R package *pROC*. In addition, a minimum value of 90% sensitivity and the highest specificity were fixed to empower the findings. CI 95% was calculated by using the Wilson score method. AUCs of ROC curves were statistically compared with the Hanley & McNeil test. Normality was evaluated with a D'Agostino & Pearson normality test and QQ-plot graphs, which showed that the dataset did not have a normal distribution (Table S3, Figure S1). Consequently, non-parametric Kruskal–Wallis test followed by Dunn's post hoc test were used to compare the different groups' pTau181 levels. Spearman correlations and simple linear regression, which included a general least squares method, were used to evaluate the correlation between CSF and plasma pTau181 levels. Cox regressions were used to evaluate the predictive value of plasma pTau181 as a proxy of AD dementia conversion in the MCI group. The start point of each survival curve was the basal visit (when CSF collection and first diagnosis were carried out), and end points were i) conversion to AD dementia for converters and ii) last follow-up visit for non-converters.

### Role of funders

Funders did not have any role in study design, data collection, data analyses, interpretation, or writing of report.

## Results

### Correlation between plasma and CSF pTau181

**Fig. 1:**
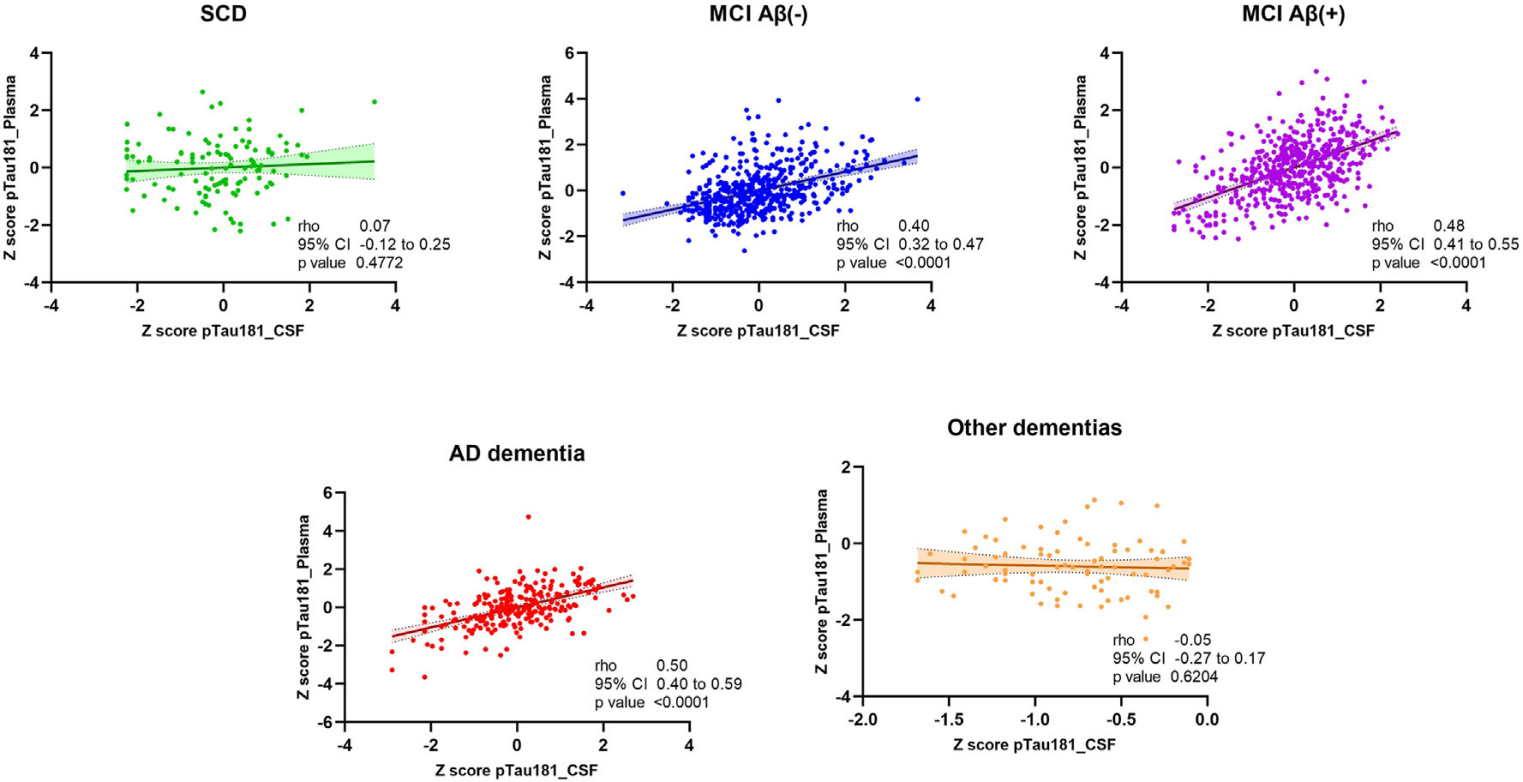
Scatter plots show the correlation between z-transformed CSF and plasma pTau181 levels in both modelling and testing cohort (n = 1628).

CSF and plasma pTau181 showed the highest correlation in both MCI Aβ(+) and AD dementia, whereas did not show correlation in SCD (rho = 0.07 [−0.12 to 0.25], p = 0.4772) and other dementias (rho = −0.05 [−0.27 to 0.17], p = 0.6204) (Fig. 1, Table S4). The comparison of the two CSF analytical techniques showed that this parameter did not affect to the CSF/plasma pTau181 correlation results (Table S3).

### Distribution of plasma pTau181 among the AD continuum

**Fig. 2:**
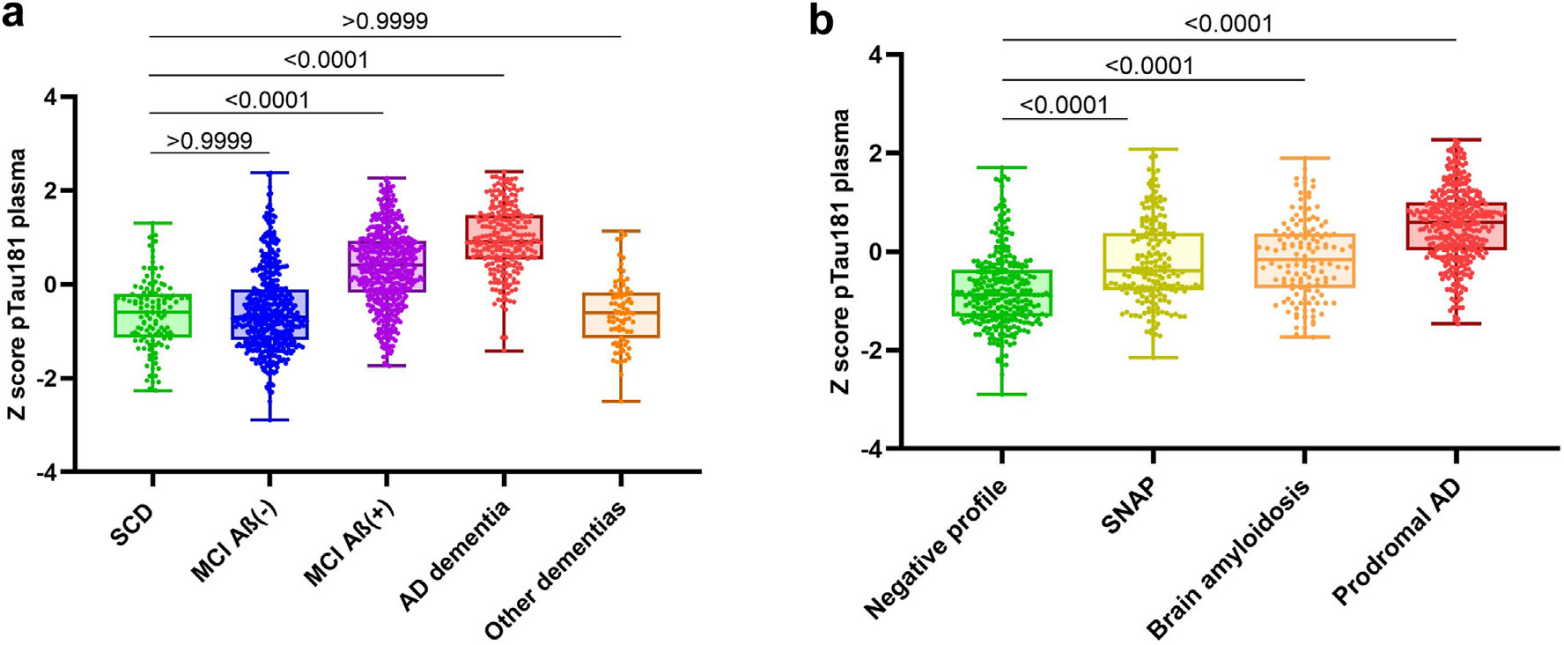
Comparison of plasma pTau181 levels among **a)** the different phenotypes of the whole study and **b)** MCI sub-groups. Non-parametric Kruskal–Wallis test. Statistic values correspond to adjusted p Value. SNAP, suspected non amyloidosis profile. #Prodromal AD is referred to those subjects with MCI due to AD.

The distribution of paired CSF/plasma pTau181 levels showed well-differentiated clusters of samples when stratified by the syndromic status along the AD continuum (Fig. 2A). As expected, plasma pTau181 levels appeared to be higher in the AD dementia group (2.70 ± 0.09 pg/ml), whereas SCD exhibited the lowest value (1.29 ± 0.08 pg/ml). MCI Aβ(+) and AD dementia showed a statistically significant difference in plasma pTau181 levels compared with the healthiest population (p < 0.0001 in both cases), not instead MCI Aβ(−) as well as other dementias subjects (Fig. 2A, Table S5). When comparing patients with MCI Aβ(+) and Aβ(−), a log-z transformed mean rank difference of 393.21 (p < 0.0001) was observed (Table S5). The comparison of AD dementia to other dementia groups showed a log-z transformed mean rank difference of 647.13 (p < 0.0001) (Table S5). Likewise, all subjects with MCI with at least one positive biomarker (suspected non-amyloidosis profile [SNAP], brain amyloidosis, and prodromal AD) showed increased levels of plasma pTau181 compared to those subjects with MCI and a negative profile. Plasma pTau181 levels of subjects with MCI with SNAP and brain amyloidosis did not exhibit any differences (Fig. 2B, Table S5).

### Influence of clinical variables on the plasma pTau181 distribution

**Fig. 3:**
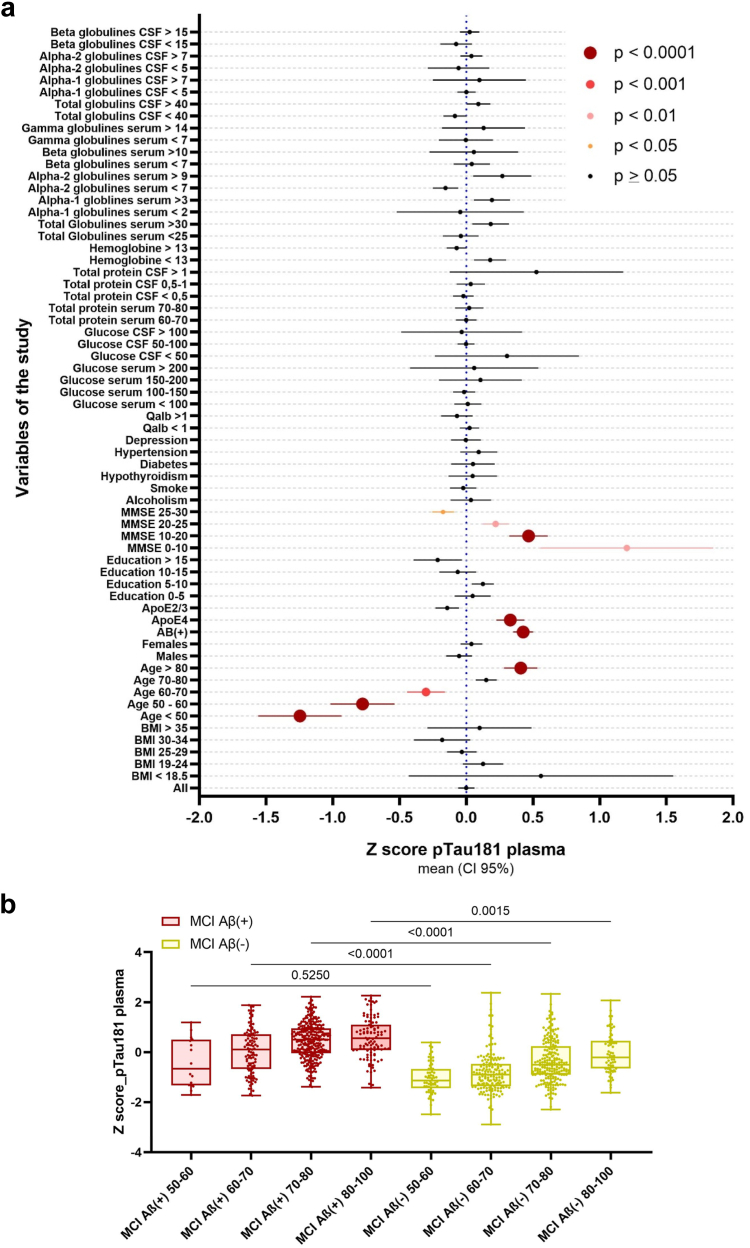
**a)** Contribution of different clinical variables in plasma pTau181 performance. Naïve plasma pTau181 (“all” group) was set as reference. **b)** Comparison of plasma pTau181 levels between Aβ(+/−). Patients with MCI stratified by age. Statistic values correspond to p Value.

Subjects were grouped by the outcomes of different clinical variables, and their plasma pTau181 levels were compared with those of the whole cohort. Of all the tested variables, age, positive amyloidosis, *APOE* ε4 presence, and cognitive status significantly correlated with the plasma pTau181 levels, which appeared to increase with positive amyloidosis, *APOE* ε4 presence, age above 70 years, and an MMSE score below 25 (Fig. 3A, Table S6). There was no difference between the sexes (p > 0.9999). When clustered by phenotypes, the AD dementia and other dementia groups did not show any significant variable influencing pTau181 levels, while in subjects with SCD, plasma pTau181 was affected only by age (p < 0.0001) (Figure S2). Although the plasma pTau181 levels of the age stratum of 70–80 years did not show differences in the full cohort (p = 0.2302), in patients with MCI stratified by age, plasma pTau181 showed increased levels in subjects with MCI Aβ(+) older than 60 years (Fig. 3B, Table S7).

### ROC curves and cut-off values

**Fig. 4:**
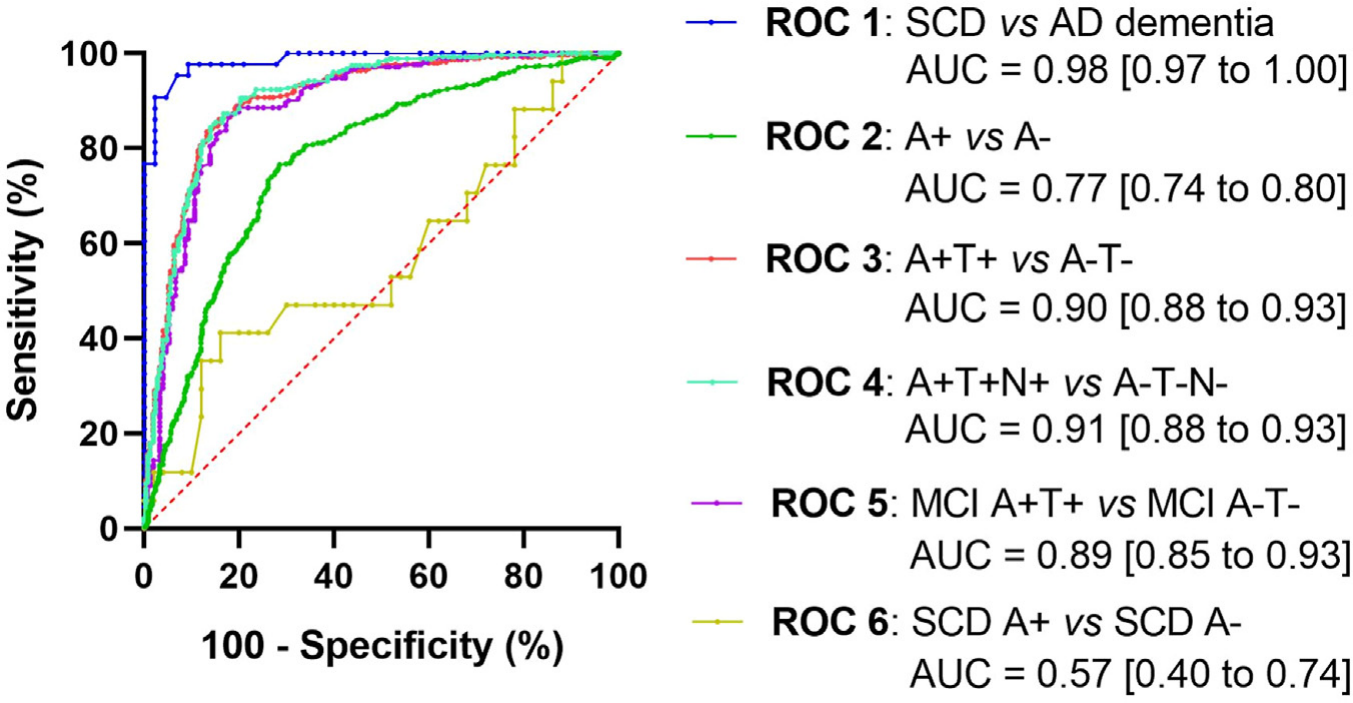
ROC curves developed from the modelling cohort to stablish the optimal cut off value for plasma pTau181.

Different combinations of case/control subjects were selected in the modelling cohort to examine the ROC curves and determined the most valuable cut off (Fig. 4). Statistical comparison of AUC is shown in Table S9. In a first step, a pilot study with specific selected subjects from the extremes of the AD continuum (SCD *vs* AD dementia) were carried out. Obtained results showed a cut off value of 2.10 pg/ml, with a sensitivity of 97.67% [87.94–99.88], a specificity of 90.70% [78.40–96.32], Youden Index of 88.37% and a ROC AUC = 0.98 [0.97–1.00], p < 0.0001 (Table 3, Table S8). These scores strongly decreased when extrapolated the cut off to real world case/control individuals, being the sensitivity lower than 66% in all the analysis (Table 3).

**Table 3:** Cut offs analysis of plasma pTau181 in the modelling cohort.

Pilot study			
SCD (A-T-N-) vs AD dementia (A + T + N+)	Sensitivity [95% CI] (%)	Specificity [95% CI] (%)	Youden index (%)
>2.10	97.67 [87.94–99.88]	90.70 [78.40–96.32]	88.37
**Real world**			
**A+ vs A-**			
>2.10	53.05 [48.84–57.22]	83.88 [80.23–86.96]	36.93
>1.03^a^	95.38 [93.27–96.85]	25.27 [21.51–29.44]	20.65
**A+T+ vs A-T-**			
>2.10	65.80 [60.94–70.36]	91.99 [88.26–94.60]	57.79
>1.34^a^	95.08 [92.44–96.83]	61.67 [55.93–67.11]	56.75
**A + T+N+ vs A-T-N-**			
>2.10	66.06 [60.30–71.39]	91.91 [88.06–94.60]	57.50
>1.36^a^	95.31 [92.14–97.24]	62.13 [56.24–67.69]	57.11
**MCI A+T+ vs A-T-**			
>2.10	59.52 [52.77–65.93]	91.39 [85.83–94.90]	50.91
>1.30^a^	94.63 [90.69–96.99]	61.49 [54.32–69.06]	56.12

a Optimal cut off value for each case/control evaluation (sensitivity ∼ 95.00%).

In the real world scenario, A + T + N + *vs* A-T-N- case/control comparison exhibited the best fit values (AUC = 0.91 [0.88–0.93], p < 0.0001) with a cut off value of 1.36 pg/ml, a sensitivity 95.31% [92.14–97.24], specificity of 62.13% [56.24–67.69] and Youden index of 57.11% (Table 2, Fig. 4). A specific ROC curve for real world subjects with MCI showed a similar performance (AUC = 0.89 [0.85–0.93], p < 0.0001) with a cut off value of 1.30 pg/ml, a sensitivity 94.63% [90.69–96.99], specificity 61.49 [54.32–69.06], and Youden index 56.12% (Table 2, Fig. 4). As expected, a specific ROC curve for subjects with SCD showed a very modest AUC (AUC = 0.57 [0.40–0.74], p = 0.3834) (Fig. 4, Table S8). Thus, specific MCI cut off of 1.30 pg/ml was selected to test the clinical relevance of the plasma pTau181 to detect prodromal AD in the context of MCI.

By applying this cut off to the testing cohort, plasma pTau181 was also able to discriminate between subjects with prodromal and non-prodromal MCI with a similar sensitivity (93.57% [88.72–96.52]) and improved specificity (72.38% [62.51–79.01]), positive prediction value (PPV, 77.85% [70.61–83.54]) and negative prediction value (NPV, 91.67% [86.28–95.08]) were also observed (Table 4). In the validation cohort, similar results were observed in the GR@CE/DEGESCO subgroup, whereas SPIN subjects exhibited a significantly reduction of the specificity (10.91% [5.23–20.31]), PPV (46.36% [37.32–56.24]) and Youden Index (10.87%) (Table 4).

**Table 4:** Characteristics of the selected plasma pTau181 cut off (1.30 pg/ml) in the of the Modelling, Testing and Validation cohorts.

∗Subjects with MCI A+T+ *vs* MCI A-T- were selected for comparisons.

### Plasma pTau181 predicts conversion in subjects with MCI

**Fig. 5:**
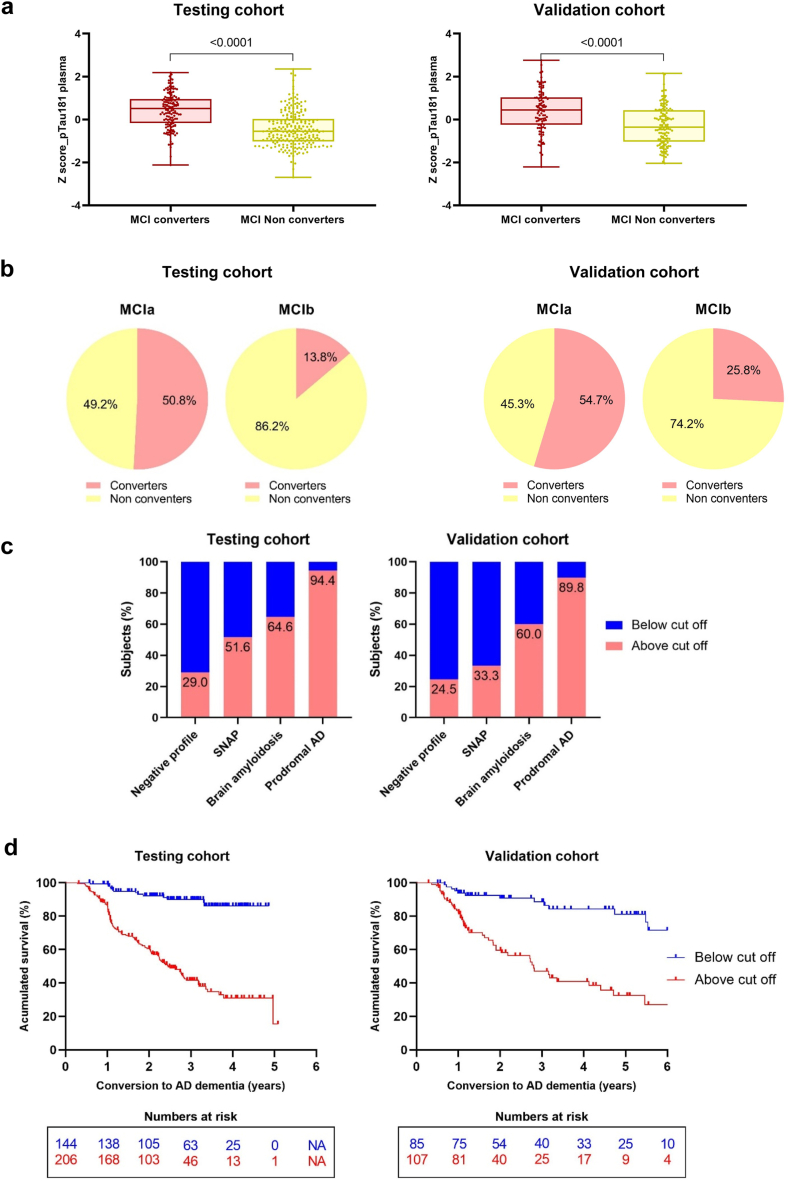
Plasma pTau181 results of conversion in the population with MCI. **a)** Differences of plasma pTau181 levels between converters and non-converters subjects with MCI. **b)** Rates of conversion of the subjects with MCI clustered by plasma pTau181 cut off (1.30 pg/ml). **c)** Proportions of subjects with MCIa and MCIb in all the MCI subgroups. **d)** Survival curves from non-adjusted cox regression analysis of MCI conversion to AD dementia grouped by plasma pTau181 cut off value (1.30 pg/ml). Follow up data from the validation cohort belong to GR@CE/DESCO cohort. “Accumulated survival” refers to the time until conversion to dementia. ∗MCIa, subjects with MCI and plasma pTau181 levels above the cut off value; MCIb, subjects with MCI and plasma pTau181 levels below the cut off value. SNAP, suspected non amyloidosis profile. ^#^Prodromal AD is referred to those subjects with MCI due to AD.

The selected cut off from the modelling cohort (1.30 pg/ml) was used in the population with MCI of the testing and validation cohorts with follow-up data, to evaluate the ability of plasma pTau181 to predict the MCI conversion to dementia. Subjects with MCI who converted to dementia exhibited higher plasma pTau181 levels than non-converters in both testing and validation cohort (p < 0.0001) (Fig. 5A, Table S10).

Regarding the conversion rate, 86.6% of the subjects with MCI from the testing cohort with plasma pTau181 levels below the cut off value (MCIb) did not convert to dementia, whereas 50.8% of those with plasma pTau181 levels above the cut off value (MCIa) did so. These scores slightly variated in the validation cohort (74.2 and 54.7% respectively) (Fig. 5B). When stratified by subgroups, the percentage of subjects with MCIa increased as the positivity of AT(N) biomarkers did too, being the prodromal AD subgroup those with the highest percentage of MCIa (94.4 and 89.8% in the testing and validation cohort, respectively) (Fig. 5C).

Finally, progression to conversion was analysed. The accumulated survival was used to refer to the time until conversion to AD dementia. Cox regressions were analysed with both simple and age-sex adjusted models, showing no difference (Table 5). The modelling cohort's results revealed that individuals with MCIa had an increased conversion rate to AD dementia compared with those with MCIb (estimate = 0.61, p = 0.0325), whose accumulated survival was shown to be stable (75.8%) at 5 years of follow-up (Fig. 5D). MCIa subjects showed an 84% higher risk of conversion than MCIb subjects (HR = 1.84 [1.05–3.22]) (Table 5). These findings were corroborated in both testing and validation cohorts (Fig. 5D–Table 5). Survival curves were statistically compared, and meta-analysis revealed non heterogeneity among curves (I^2^ = 0%, X^2^ = 0.22, p = 0.8951).

**Table 5:** Cox regression results of the survival analysis of the subjects with MCI of the modelling, testing and validation cohorts.

∗Graph correspond to: blue = HR of Cox analysis by order; yellow/red = HR of meta-analysis by order.

## Discussion

In this study we evaluated the clinical applicability of plasma pTau181 in the CoU of screening patients in the primary care centres and memory clinics with a suspected high risk of developing AD dementia. The study was designed following the latest recommendations of the Alzheimer's Association for the translation of plasma biomarkers to the clinical practice,^5^ and included one of the largest real-world cohorts with both CSF/plasma data reported to date. Importantly, we modelled, tested and validated the results in three fully independent cohorts.

Our results confirmed that plasma pTau181 correlated with CSF pTau181 in both MCI and AD dementia, in agreement with previous studies,^10^^,^^20^^,^^36^ but not in subjects with SCD and other dementias, which highlights the AD specificity of pTau181. Regarding SCD, this finding could be due to the early stage of the SCD phase in the disease continuum, in which the CNS levels are not high enough for their correlational translation to plasma, as well as the potential confounding effect of age on the measurements. In addition, this could be also due to the discrepancies between CSF biomarkers and PET scans in this early stage (since most plasma biomarkers have been developed with PET as the gold standard), which change AT(N) status. We repeated this analysis with both PET scan and CSF biomarkers in a small subset of 68 patients with SCD. It was shown that, although plasma pTau181 ROC results were improved with PET subjects’ stratification (AUC = 0.74 [0.61–0.87] *vs* 0.57 [0.40–0.74] respectively), they were still not specific enough to distinguish the Aβ status of participants with SCD (specificity: 56.25 *vs* 22.00%). The improvement was insufficiently robust to conclude that plasma pTau181 had a predictive value in SCD (Figure S3).

Regarding the plasma pTau181 levels, our study revealed that this biomarker appeared to increase along the disease continuum, which was also in agreement with previous findings.^19^^,^^21^^,^^22^ In this sense, several studies have highlighted the robust clinical performance of pTau181 to differentiate AD dementia, with a well-established Aβ(+) status, from Aβ(−) controls.^37^^,^^38^ In our hands, subjects with MCI Aβ(+) showed increased levels of plasma pTau181 compared to those subjects with MCI Aβ(−). The same results were found between subjects with AD dementia and other dementias. The distribution of plasma pTau181 levels showed differences when clustered by age, APOE ε4 status, and an MMSE <25, whereas sex did not, which was in agreement with those published by Baiardi et al. In preliminary analysis, the models were adjusted by age and sex (the most common adjustments). Sex did not have any effect, while age did. However, all the correlations and differences between age groups disappeared, suggesting that part of the plasma pTau181 performance is conducted by age. In contrast, Cox regression showed no differences with the simplest model when adjusted by sex and age. For these reasons, and to avoid the overfitting effects, we do not recommend adjusting the analysis.

When evaluated the plasma pTau181 in the pilot case–control study, a cut off value of 2.10 pg/ml, with more than both 90% of sensitivity and specificity, was found. These values were in agreement with the results reported by Arranz et al., who analysed the performance of plasma pTau181 with the same analytic platform and obtained a cut off value of 2.01 pg/ml.^32^ However, when transferred this cut off to the real-world subjects, these parameters sharply dropped. Instead, the specific cut off for real-world individuals with MCI (1.30 pg/ml) was able to differentiate between MCI subjects with prodromal and non-prodromal AD in the testing cohort with great sensitivity and specificity results. Studies carried out by Janelidze et al.^39^ and Smirnov et al.^40^ showed similar results, but the small number of Aβ(+) subjects of these studies and the selection bias of included patients limited the precision and translation of obtained cut off to the real-world. Testing cohort results were confirmed in the GR@CE/DEGESCO validation cohort, but not in the SPIN cohort. This may be due to clinical differences between cohorts, since SPIN cohort showed increased mean plasma pTau181 levels compared to GR@CE/DEGESCO cohort (2.5 *vs* 1.6 pg/ml respectively), education (11.9 vs 8.2 years respectively), or ApoE ε4ε4 (2.2 *vs* 0.7% respectively). Patients with MCI of the SPIN cohort could be in an advanced step in the dementia continuum, which is in agreement with previously reported description of the type patients who commonly arrive to the general hospital.^25^ Moreover, Aβ42 levels and A+ percentages of patients in the SPIN cohort showed a high proportion of amyloidosis. The definition of the cut-off values for AT(N) classification in each centre (ACE and San Pau Hospital) could contribute to this bias. Furthermore, it has already been described how variability and bias of blood measurements in multicentre studies can affect plasma biomarkers’ quantification.^41^, ^42^, ^43^ The existence of pre-analytical differences in sample collection, batches of kits and reagents, storage time, or inherent differences between equips and technical personnel could also explain these discrepancies. All this together suggests that cut-off values cannot be directly implemented in other centres without validation.

One of the most relevant findings of this real-world study was the ability of plasma pTau181 to predict the conversion from MCI to AD dementia, which is extremely valuable in the clinical routine. Subjects with MCI who converted to AD dementia exhibited higher values of plasma pTau181 levels than non-converters MCI in both testing and validation cohorts. Moreover, around 80% of the subjects with MCIb in both testing and validation cohorts did not convert to AD dementia during the mean five years of follow-up. In addition, survivals curves showed that subjects with MCIa exhibited a dramatic high rate of AD dementia conversion, with 84% of higher risk of conversion to AD dementia than those individuals with MCIb. These findings were corroborated in both testing and validation cohorts. Although the HR has been called into question since it may change over time and has a built-in selection bias,^44^ its agreement with the survival curves support this finding.

These results agree with a previous study from Planche et al., which reported that blood pTau181 alone was the best blood predictor of the 5-year AD/mixed dementia risk.^22^ Similarly, Palmqvist et al. showed that a model combining plasma pTau181, memory, executive function, and APOE produced the highest accuracy (AUC = 0.90, p < 0.001) to differentiate future AD dementia in participants with SCD and MCI from the BioFINDER (n = 340) and ADNI (n = 543) cohorts.^45^ In this sense, Sarto et al. also evaluated the diagnostic performance and clinical applicability of different blood pTau181 in a prospective cohort of the Hospital Clinic Barcelona, Spain.^46^ It was found that plasma pTau181 had a high diagnostic accuracy for differentiating AD from non-neurodegenerative causes (AUC = 0.94) and predicted amyloid status (85% sensitivity and specificity) with an accurate individual precision in approximately 60% of the patients.^46^ However, some biases can affect the interpretation and robustness of this study, such as the low number of participants with reference AD biomarkers, the young mean age of the cohort (65 years, which may not be valid for real-world memory clinic populations), or some underpowered diagnostic categories represented (i.e., CJD, DLB).

Finally, although this study was conducted with more than 1800 CSF/plasma paired samples in a real-world clinical cohort, a huge clinical dataset, and three independent cohorts, our study also has some limitations. Firstly, due to the complexity of finding patients with SCD and the ethical restrictions on performing a lumbar puncture, most of this population came from the FACEBHI and BIOFACE research studies, which could contribute to a potential selection bias. In addition, the number of participants with SCD was lower than the number of participants in the other groups of the study, and due to the healthier condition of this population, the number of Aβ(+) patients was somewhat low. Since renal function has been shown to contribute to variations in the levels of some plasma biomarkers, the lack of this information in our clinical data could also represent some limitations to fully understanding the performance of plasma pTau181. Unfortunately, we do not perform the AT(N) classification with PET scans. It is unaffordable for a memory clinic's routine, mainly due to its elevated cost, but also because of its invasive nature, and risk of radioactivity exposure. Therefore, we had to use CSF as the gold standard reference. About the validation cohort, not all the subjects had CSF biomarkers, which could contribute to already reported lower accuracy in the clinical diagnosis by memory clinic physicians.^45^ Moreover, the reduced number of subjects of the validation cohort, as well as the clinical bias inherent to the origin of the cohorts, all together could explain the differences observed when compared to the modelling and testing cohorts. Finally, this study did not have longitudinal measurements of plasma pTau181, so it could not be evaluated the changes in the biomarker over time.

In conclusion, outstanding challenges, such as the validation of findings in other independent cohorts, a standardization of laboratory analytics, or a universal guideline for their clinical implementation should be addressed in the upcoming years to enable a robust progress of plasma biomarkers in different CoUs. Plasma pTaus have shown better results than Aβs in recent years, becoming a promising biomarker for AD screening.^21^ Different pTau isoforms have shown great results for different aims and disease continuum stages. In this sense, plasma pTau217 has been shown to be highly accurate in the earliest stages and be representative of the longitudinal progression of the disease.^21^ Plasma pTau231 is abnormally increased in the preclinical phase, even before the AB PET threshold becomes positive.^15^^,^^47^ In contrast, pTau181, the gold standard used in CSF AT(N) classification, has been shown to be highly specific to AD dementia, allowing us to discriminate AD from other dementias and discriminate between AB ± PET scans.^19^ Moreover, plasma pTau has also been shown to be highly specific for post-mortem confirmation of AD pathology.^23^ Our results demonstrate that plasma pTau181 has real potential to detect AD pathology in subjects with MCI. We propose its use in this CoU and recommend the validation of internal cut-offs of plasma biomarkers in each centre to ensure their proper diagnostic performance. Based on current results, and until more large-scale real-world studies corroborate these findings, we consider that plasma pTau181 positivity still requires an AD pathology confirmatory test (PET or CSF) using the gold-standard diagnosis protocols. Likewise, previous studies with plasma pTau217 have explored a two-step workflow study design to screen for Aβ positivity, which could also improve the results obtained for plasma pTau181.^48^ Plasma pTau181 by itself does not represent a suitable biomarker for detecting AD pathology in patients with SCD, so another biomarker or a combination of biomarkers would be needed to address AD pathology in healthy populations. SCD phenotype represents one of the greatest challenges for plasma biomarker development, not only because of the identification of these patients without any clinical symptoms and the importance of the pre-clinical phase in the beginning of the molecular alterations of the brain but also because of the difficulty of detecting them in the periphery. Ultimately, larger studies such as the one described here, are needed to corroborate these indications and confirm these results. However, our study confirms that plasma pTau181 represents a promising tool as screening biomarker of AD pathology in a real-world population with MCI.

## Contributors

All authors have read and approved the manuscript. Amanda Cano designed and conceptualised the study, performed the experimental assays, analysed and interpreted the data, designed the figures and tables, and wrote the manuscript. María Capdevila, Raquel Puerta, and Laura Montrreal contributed to the experimental assays, data acquisition and manuscript revision. Javier José Arranz-Martínez, Alberto Lleó, Juan Fortea and Daniel Alcolea contributed to the experimental assays and data acquisition of the SPIN cohort, and manuscript revision. Itziar de Rojas, Pablo García-González, Claudia Olivé, Fernando García-Gutiérrez, Oscar Sotolongo-Grau, Adelina Orellana, Nuria Aguilera, Maribel Ramis, Maitee Rosende-Roca, Juan Pablo Tartari, Asunción Lafuente, Liliana Vargas, Alba Pérez-Cordón, Nathalia Muñoz, Ángela Sanabria, Montserrat Alegret, Xavier Morató, Lluís Tárraga, Victoria Fernández, Marta Marquié, and Sergi Valero, contributed to data acquisition, interpretation and manuscript revision. Mercè Boada contributed to the supervision of the study, obtaining the financing support and manuscript revision. Agustín Ruíz contributed to the conceptualization, supervision, data interpretation, writing of the manuscript and obtaining the financing support. A Cano, M. Marquié, M. Boada and A. Ruíz have verified the underlying data.

## Data sharing statement

The data that support the findings of this study are available from the corresponding authors upon a reasonable request and a data transfer agreement.

## Declaration of interests

Alberto Lleó received personal fees for service on the advisory boards from Biogen, Eisai, Fujirebio-Europe, Lilly, Novartis, NovoNordisk, Nutricia, Otsuka Pharmaceutical, and Zambón, and received speaker honoraria from Lilly, Biogen, KRKA and Zambon.

Juan Fortea received personal fees for service on the advisory boards, adjudication committees or speaker honoraria from AC Immune, Lilly, Lundbeck, Roche, Fujirebio and Biogen, outside the submitted work. D.A., A.L. and J.F. report holding a patent for markers of synaptopathy in neurodegenerative disease (licensed to Adx, EPI8382175.0).

Marta Marquié received personal fees for service on the advisory boards from Araclon Biotech–Grifols, S.A. Marta Marquié received grants or contracts from Instituto de Salud Carlos III (ISCIII) Accion Estrategica en Salud, integrated in the Spanish National RCDCI Plan and financed by ISCIII-Subdireccion General de Evaluacion and the Fondo Europeo de Desarrollo Regional (FEDER—Una manera de hacer Europa) grant PI19/00335.

Daniel Alcolea received personal fees for service on the advisory boards from Fujirebio-Europe, Roche Diagnostics, Nutricia, Krka Farmacéutica S.L., Zambon S.A.U., Grifols, S.A., Lilly, and Esteve Pharmaceuticals S.A.
