# Supplementary material for: Clinical value of plasma pTau181 to predict Alzheimer's disease pathology in a large real-world cohort of a memory clinic
Source: eBioMedicine. 2024 Sep 18;108:105345. doi: 10.1016/j.ebiom.2024.105345 (PMC11424964; doi:10.1016/j.ebiom.2024.105345)
Supplement: Supplementary Material, Figures and Tables [file mmc1.pdf]

Supplementary material

Clinical value of plasma pTau181 to predict Alzheimer's disease pathology in a large real-world cohort of a memory clinic

Cano *et al*. 2024

2. Methods

2.2 Participants, study groups and selection criteria.

All subjects were examined with the Spanish version of the Mini-Mental State Examination (MMSE) (1,2), the memory part of the Spanish version of the 7 Minute test (3), the Spanish version of the Neuropsychiatric Inventory Questionnaire (NPI-Q)(4), the Hachinski Ischemia Scale(5), the Blessed Dementia Scale (6), the Clinical Dementia Rating (CDR) scale (7) and a comprehensive neuropsychological battery of Ace (N-BACE) (8,9).

The subjective cognitive decline (SCD) syndromic diagnosis is given to individuals with subjective perception of memory loss or other cognitive problems without any objective evidence of impairment when evaluated with standardized cognitive tests (10).

The mild cognitive impairment (MCI) syndromic diagnosis is given to patients showing deficits in one or more cognitive domains on formal neuropsychological testing according to age and educational level (assessed by the NBACE (8)) but with a full preserved autonomy in daily activities. In addition, this MCI group was further classified as Amnestic *vs* Non-amnestic and Possible *vs* Probable subtypes (11). An Amnestic MCI subtype was assigned when memory deficits were present, while a Non-amnestic MCI subtype was used to classify patients who presented with preserved memory but deficits in other cognitive domains (12). The Possible or Probable MCI status refers to the presence or absence, respectively, of comorbidities (such as cerebrovascular pathology, psychiatric and systemic disorders) that could explain or contribute to the cognitive deficits (13,14). Regarding the MCI longitudinal cohort, CSF results with associated follow-up data were available from 388 and 374 MCI patients in the testing and validation cohorts, respectively. Patients with MCI were evaluated and followed up at a single site (ACE). Baseline and follow-up data were obtained between 2016 and 2022. All participants with MCI were assessed as previously reported, and follow-up assessments were conducted on an approximately annual basis. The MMSE and NBACE batteries were measured at all visits. Dementia conversion was defined using previously published criteria (15).

Finally, the dementia syndromic diagnosis is defined according to the DSM-V criteria (16). The underlying aetiologies within dementia groups were classified according to the following criteria. The National Institute on Aging and Alzheimer's Association (NIA-AA) was used for Alzheimer´s disease (AD) (17), and AD pathology was defined as an A+T+N(+/-) classification and a syndromic diagnosis of dementia. The National Institute of Neurological Disorder and Stroke and the Association Internationale pour la Recherche et l'Enseignement in Neurosciences criteria (NINDS-AIREN) were used for vascular dementia (VD) (18), frontotemporal dementia (FTD) (19), and Lewy body

dementia (LBD) (20). The main diagnoses of the participants with non-AD dementia were vascular, Lewy bodies and frontotemporal dementia.

Figures

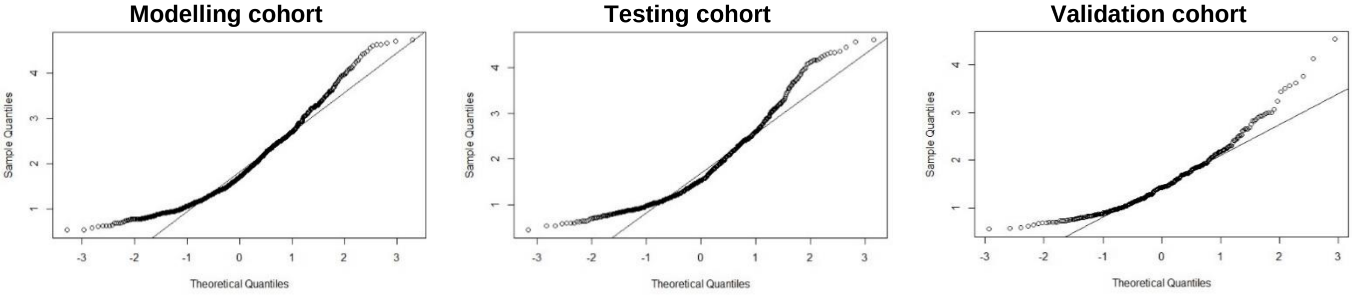

Figure S1. QQ plots of the normality tests of the modelling, testing and validation cohorts of the study.

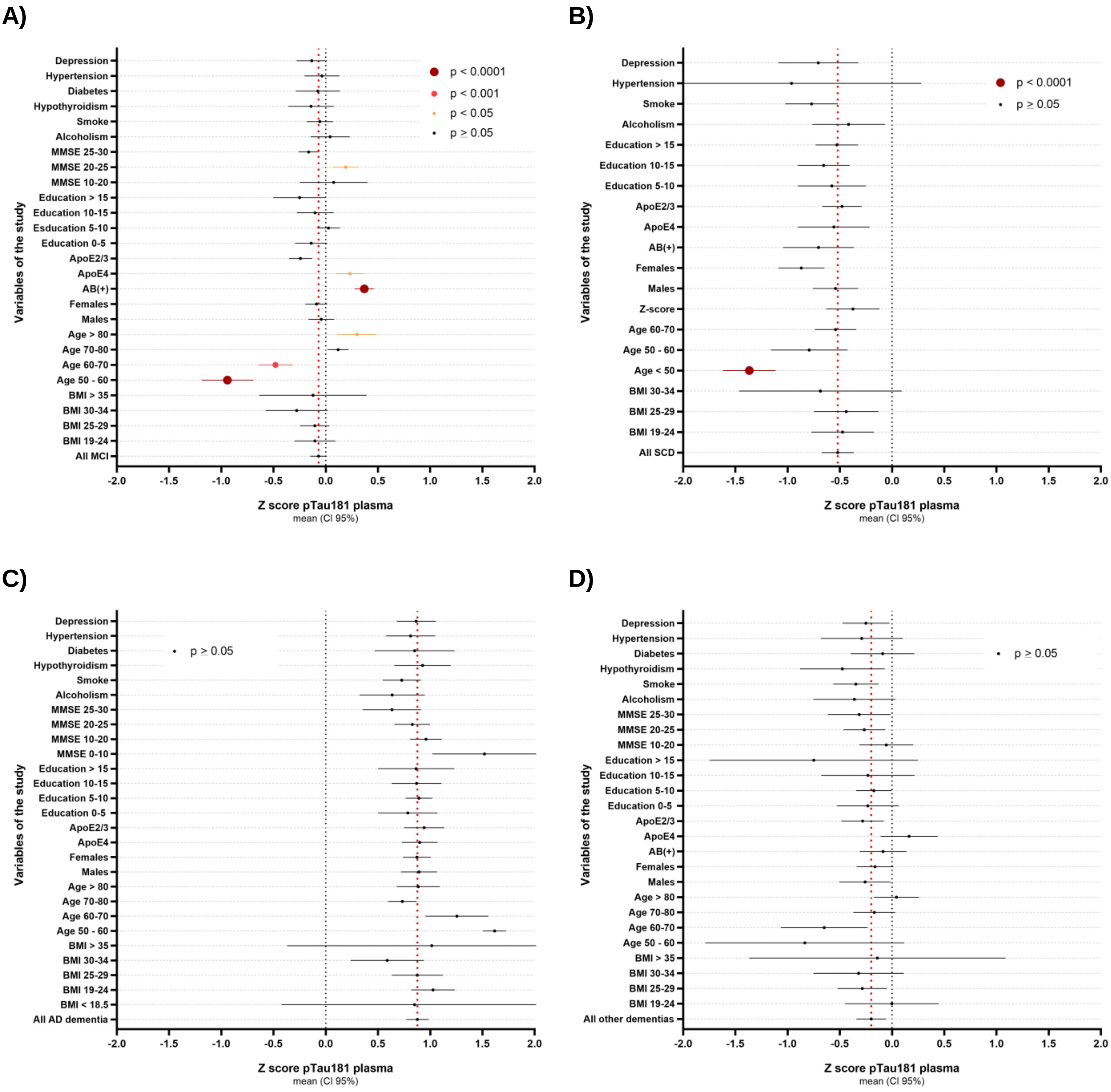

Figure S2. Influence of clinical variables in plasma pTau181 distribution in **A)** patients with MCI, **B)** patients with SCD, **C)** patients with AD dementia, and **D)** patients with other dementias.

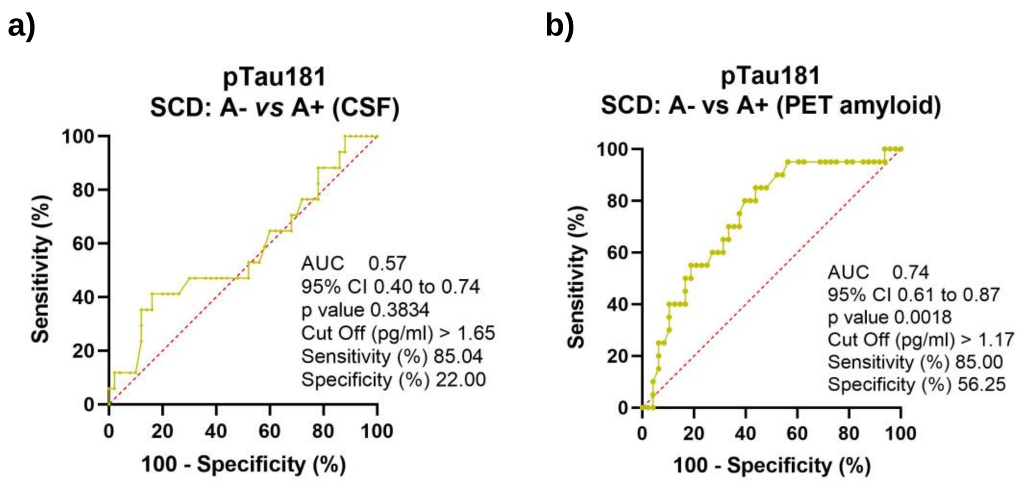

Figure S3. Comparison of plasma pTau181 ROC curves of SCD Aβ(+) vs Aβ(-) subjects stratified by **A)** CSF Aβ42 biomarker and **B)** PET scan centiloid (cut off for early amyloid deposition set as 13.5 centiloids as described by Pascual-Lucas *et al.* 2023 (21).

Tables

Table S1. Percentage of the studied clinical variables in the modelling, testing and validation cohorts.

Cohorts of the study	Modelling	Testing	Validation
**Variables**	**(%)**	**(%)**	**(%)**
Age < 50 years	2.63	1.10	0.76
Age > 80 years	18.20	18.74	9.92
Age 50 – 60 years	6.27	8.98	9.16
Age 60 – 70 years	22.04	28.50	19.85
Age 70 – 80 years	50.86	42.68	60.31
Alcoholism	10.50	9.46	10.14
Alpha-1 globulins CSF < 5 g/L	83.72	80.44	97.71
Alpha-1 globulins CSF > 7 g/L	3.84	6.15	0.00
Alpha-1 globulins serum < 2 g/L	2.73	3.63	2.29
Alpha-1 globulins serum > 3 g/L	28.74	22.87	13.74
Alpha-2 globulins CSF < 5 g/L	9.20	8.36	3.82
Alpha-2 globulins CSF > 7 g/L	61.78	68.93	61.83
Alpha-2 globulins serum < 7 g/L	45.55	50.00	45.04
Alpha-2 globulins serum > 9 g/L	8.50	5.99	6.11
ApoE2/3	65.69	64.92	66.39
ApoE4	34.31	35.08	33.61
Aβ42(+)	53.95	52.52	54.20
Beta globulins CSF < 15 g/L	27.91	21.92	49.62
Beta globulins CSF > 15 g/L	72.60	79.18	51.91
Beta globulins serum < 7 g/L	22.17	27.92	25.19
Beta globulins serum >10 g/L	4.05	4.26	3.82
BMI < 18.5 kg/m2	0.87	0.17	0.00
BMI > 35 kg/m2	3.69	4.50	5.43
BMI 19-24 kg/m2	34.74	30.67	37.21
BMI 25-29 kg/m2	47.12	48.83	41.09
BMI 30-34 kg/m2	13.57	15.83	16.28
Depression	34.78	33.84	33.08
Diabetes	16.86	12.75	13.85

Education >15 years	11.44	8.80	11.45
Education 0-5 years	20.99	17.44	12.98
Education 10-15 years	13.85	17.60	19.85
Education 5-10 years	53.73	56.16	55.73
Females	59.76	56.45	59.54
Gamma globulins serum < 7 g/L	9.92	9.31	4.58
Gamma globulins serum > 14 g/L	4.25	3.63	3.05
Glucose CSF < 50 mg/dL	1.31	1.58	0.00
Glucose CSF >100 mg/dL	2.33	4.26	3.05
Glucose CSF 50-100 mg/dL	96.36	94.16	96.95
Glucose serum < 100 mg/dL	42.09	41.01	48.09
Glucose serum >200 mg/dL	1.72	1.74	0.76
Glucose serum 100-150 mg/dL	52.03	52.52	45.80
Glucose serum 150-200 mg/dL	4.16	4.73	5.34
Haemoglobin < 13 g/dL	29.98	28.55	38.17
Haemoglobin > 13 g/dL	70.02	71.45	61.83
Hypertension	48.56	54.50	50.47
Hypothyroidism	12.57	10.10	14.62
Males	40.24	43.55	40.46
MMSE 0-10	0.97	0.33	0.00
MMSE 10-20	17.63	14.26	9.30
MMSE 20-25	31.40	32.34	20.93
MMSE 25-30	50.00	53.07	69.77
Qalb <1	92.60	92.43	89.31
Qalb >1	7.40	7.57	10.69
Smoke	27.07	23.89	28.81
Total Globulins serum <25 g/L	20.55	23.50	22.14
Total Globulins serum >30 g/L	20.45	20.50	18.32
Total globulins CSF < 40 g/L	50.66	39.43	80.92
Total globulins CSF > 40 g/L	49.34	60.57	19.08
Total protein CSF < 0.5 g/L	67.34	66.88	72.52
Total protein CSF > 1 g/L	1.11	1.42	0.76
Total protein CSF 0.5-1 g/L	31.55	31.70	26.72
Total protein serum 60-70 g/L	59.51	61.61	67.18
Total protein serum 70-80 g/L	38.26	36.49	32.06

* Data only available in the GR@CE/DEGESCO cohort.

Table S2. Sensitivity analysis of the outlier's removal.

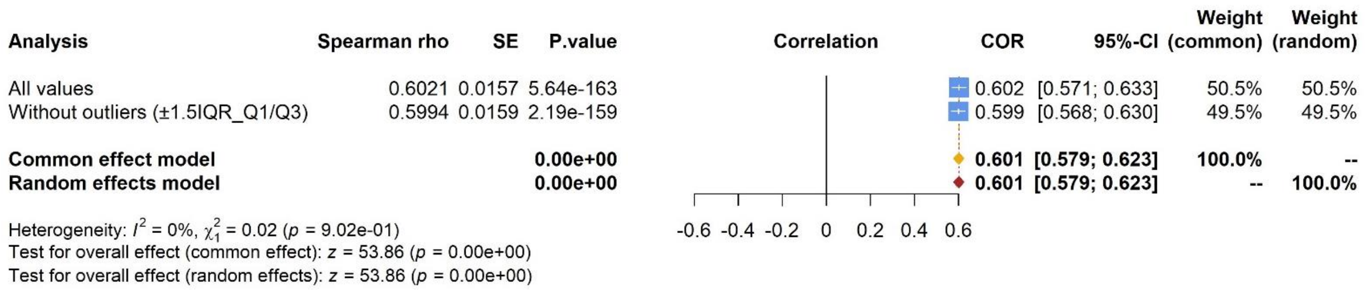

Table S3. Results of the D'Agostino & Pearson normality test in the modelling, testing and validation cohorts.

Normality test	Modelling cohort	Testing cohort	Validation cohort
**D'Agostino & Pearson test**			
K2	47.79	27.45	7.75
p value	<0.0001	<0.0001	0.0208
Passed normality test (alpha=0.05)?	No	No	No

Table S4. Associations between log transformed z-scored CSF and plasma pTau181 among the different phenotypes of the study.

Associations between CSF and plasma pTau181 levels					
**Full cohort**	**SCD**	**MCI Aβ(-)**	**MCI Aβ(+)**	**AD dementia**	**Other dementias**
**Spearman corr.**					
rho	0.07	0.40	0.48	0.50	-0.05
95% CI	-0.12 to 0.25	0.32 to 0.47	0.41 to 0.55	0.40 to 0.59	-0.27 to 0.17
p value (two-tailed)	0.4772	<0.0001	<0.0001	<0.0001	0.6204
**Lin. Reg.**					
Slope	0.06	0.41	0.52	0.52	-0.09
95% CI (slope)	-0.10 to 0.22	0.33 to 0.49	0.45 to 0.60	0.41 to 0.63	-0.48 to 0.30
R^2	0.00	0.17	0.27	0.27	0.00
Sy.x	10.02	0.91	0.85	0.85	0.71
**Is slope significantly non-zero?**					
F	0.53	98.78	191.70	89.63	0.22
p value	0.4688	<0.0001	<0.0001	<0.0001	0.6420
**ELISA**	**SCD**	**MCI Aβ(-)**	**MCI Aβ(+)**	**AD dementia**	**Other dementias**
**Spearman corr.**					
rho	0.22	0.38	0.37	0.59	-0.03
95% CI	-0.06 to 0.47	0.25 to 0.49	0.23 to 0.50	0.47 to 0.69	-0.34 to 0.30
p value (two-tailed)	0.1119	<0.0001	<0.0001	<0.0001	0.8337
**Lin. Reg.**					
Slope	0.23	0.47	0.40	0.67	-0.12
95% CI (slope)	-0.12 to 0.58	0.31 to 0.62	0.27 to 0.53	0.51 to 0.82	-0.72 to 0.47
R^2	0.03	0.14	0.18	0.35	0.00
Sy.x	0.76	0.89	0.73	0.72	0.72
**Is slope significantly non-zero?**					
F	1.71	35.22	38.39	75.33	0.18
p value	0.1974	<0.0001	<0.0001	<0.0001	0.6713
**CLEIA**	**SCD**	**MCI Aβ(-)**	**MCI Aβ(+)**	**AD dementia**	**Other dementias**
**Spearman corr.**					
rho	0.11	0.40	0.58	0.58	0.08
95% CI	-0.15 to 0.35	0.29 to 0.50	0.50 to 0.65	0.47 to 0.68	-0.25 to 0.39
P value (two-tailed)	0.3921	<0.0001	<0.0001	<0.0001	0.6231
**Lin. Reg.**					
Slope	0.14	0.40	0.47	0.517	0.18
95% CI (slope)	-0.11 to 0.40	0.2992 to 0.4958	0.40 to 0.54	0.41 to 0.61	-0.35 to 0.71
R^2	0.02	0.19	0.34	0.37	0.01
Sy.x	0.71	0.70	0.67	0.74	0.63
**Is slope significantly non-zero?**					
F	1.25	63.37	166.70	98.35	0.49
p value	0.2675	<0.0001	<0.0001	<0.0001	0.4882

Table S5. Comparisons of log transformed z-scored plasma pTau181 levels among phenotypes in the full cohort and MCI sub-groups.

**Non-parametric Kruskal-Wallis**	**SCD**	**MCI Aβ(-)**	**MCI Aβ(+)**	**AD dementia**	**Other dementias**
Minimum	-2.26	-2.90	-1.7	-1.41	-2.49
25% Percentile	-1.14	-1.19	-0.18	0.53	-1.14
Median	-0.60	-0.69	0.41	0.91	-0.61
75% Percentile	-0.20	-0.11	0.92	1.47	-0.17
Maximum	1.30	2.38	2.27	2.40	1.14
Mean	-0.64	-0.55	0.36	0.96	-0.60
95% CI	-0.77 to-0.51	-0.63 to -0.47	0.29 to 0.43	0.87 to 1.04	-0.75 to -0.44

**Dunn's multiple comparisons test**	**Mean rank diff,**	**Adjusted p value**
SCD vs. MCI Aβ(-)	-30.95	>0.9999
SCD vs. MCI Aβ(+)	-424.20	<0.0001
SCD vs. AD dementia	-661.60	<0.0001
SCD vs. Other dementias	-14.50	>0.9999
MCI Aβ(-) vs. MCI Aβ(+)	-393.21	<0.0001
MCI Aβ(-) vs. AD dementia	-630.72	<0.0001
MCI Aβ(-) vs. Other dementias	16.45	>0.9999
MCI Aβ(+) vs. AD dementia	-237.50	<0.0001
MCI Aβ(+) vs. Other dementias	409.71	<0.0001
AD dementia vs. Other dementias	647.13	<0.0001

**Non-parametric Kruskal-Wallis**	**Negative profile**	**SNAP**	**Brain amyloidosis**	**Prodromal AD**
Minimum	-2.90	-2.15	-1.74	-1.47
25% Percentile	-1.32	-0.78	-0.74	0.03
Median	-0.88	-0.40	-0.16	0.59
75% Percentile	-0.37	0.38	0.37	1.01
Maximum	1.71	2.07	1.89	2.27
Mean	-0.79	-0.21	-0.14	0.54
95% CI	-0.88 to -0.71	-0.33 to -0.09	-0.28 to 0.00	0.46 to 0.62

**Dunn's multiple comparisons test**	**Mean rank diff,**	**Adjusted p value**
Negative profile vs. SNAP	-177.20	<0.0001
Negative profile vs. Brain amyloidosis	-202.91	<0.0001
Negative profile vs. Prodromal AD	-409.82	<0.0001
SNAP vs. Brain amyloidosis	-25.70	>0.9999
SNAP vs. Prodromal AD	-232.70	<0.0001
Brain amyloidosis vs. Prodromal AD	-207.01	<0.0001

Table S6. Clinical variables that significantly contribute to the performance of plasma pTau181

Non-parametric Kruskal-Wallis	Mean	95% CI	Adjusted p value
Dunn's multiple comparisons test			
Age < 50	-12.47	-1.56 to -0.93	<0.0001
Age 50 - 60	-0.78	-1.02 to -0.54	<0.0001
Age 60-70	-0.31	-0.44 to -0.16	0.0007
Age > 80	0.41	0.28 to 0.53	<0.0001
Aβ42(+)	0.43	0.35 to 0.50	<0.0001
ApoE4	0.33	0.22 to 0.43	<0.0001
MMSE 0-10	12.04	0.56 to 1.85	0.0296
MMSE 10-20	0.47	0.32 to 0.61	<0.0001
MMSE 20-25	0.22	0.12 to 0.32	0.0124
MMSE 25-30	-0.17	-0.25 to -0.09	0.0415

Table S7. Comparison of log transformed z-scored plasma pTau181 levels between subjects with MCI Aβ(+/-) stratified by age.

Non-parametric Kruskal-Wallis	Mean rank diff,	Adjusted p value
**Dunn's multiple comparisons test**		
MCI Aβ(+) 50-60 vs. MCI Aβ(+) 60-70	-142.13	>0.9999
MCI Aβ(+) 50-60 vs. MCI Aβ(+) 70-80	-269.51	0.0071
MCI Aβ(+) 50-60 vs. MCI Aβ(+) 80-100	-293.70	0.0040
MCI Aβ(+) 50-60 vs. MCI Aβ(-) 50-60	189.70	0.5250
MCI Aβ(+) 50-60 vs. MCI Aβ(-) 60-70	122.50	>0.9999
MCI Aβ(+) 50-60 vs. MCI Aβ(-) 70-80	-16.91	>0.9999
MCI Aβ(+) 50-60 vs. MCI Aβ(-) 80-100	-110.53	>0.9999
MCI Aβ(+) 60-70 vs. MCI Aβ(+) 70-80	-127.45	0.0031
MCI Aβ(+) 60-70 vs. MCI Aβ(+) 80-100	-151.63	0.0048
MCI Aβ(+) 60-70 vs. MCI Aβ(-) 50-60	331.72	<0.0001
MCI Aβ(+) 60-70 vs. MCI Aβ(-) 60-70	264.61	<0.0001
MCI Aβ(+) 60-70 vs. MCI Aβ(-) 70-80	125.21	0.0082
MCI Aβ(+) 60-70 vs. MCI Aβ(-) 80-100	31.61	>0.9999
MCI Aβ(+) 70-80 vs. MCI Aβ(+) 80-100	-24.20	>0.9999
MCI Aβ(+) 70-80 vs. MCI Aβ(-) 50-60	459.12	<0.0001
MCI Aβ(+) 70-80 vs. MCI Aβ(-) 60-70	392.00	<0.0001
MCI Aβ(+) 70-80 vs. MCI Aβ(-) 70-80	252.61	<0.0001
MCI Aβ(+) 70-80 vs. MCI Aβ(-) 80-100	159.01	0.0012
MCI Aβ(+) 80-100 vs. MCI Aβ(-) 50-60	483.33	<0.0001
MCI Aβ(+) 80-100 vs. MCI Aβ(-) 60-70	416.22	<0.0001
MCI Aβ(+) 80-100 vs. MCI Aβ(-) 70-80	276.81	<0.0001
MCI Aβ(+) 80-100 vs. MCI Aβ(-) 80-100	183.21	0.0015
MCI Aβ(-) 50-60 vs. MCI Aβ(-) 60-70	-67.12	>0.9999
MCI Aβ(-) 50-60 vs. MCI Aβ(-) 70-80	-206.52	<0.0001
MCI Aβ(-) 50-60 vs. MCI Aβ(-) 80-100	-300.14	<0.0001
MCI Aβ(-) 60-70 vs. MCI Aβ(-) 70-80	-139.45	0.0001
MCI Aβ(-) 60-70 vs. MCI Aβ(-) 80-100	-233.02	<0.0001
MCI Aβ(-) 70-80 vs. MCI Aβ(-) 80-100	-93.62	0.5651

Table S8. Characteristics of the ROC curves in the Modelling cohort.

	ROC 1	ROC 2	ROC 3	ROC 4	ROC 5	ROC 6
**Category**	**Pilot**	**Real World**	**Real World**	**Real World**	**Real World**	**Real World**
**Controls**	**SCD**	**A-**	**A-T-**	**A-T-N-**	**MCI A-T-**	**SCD A-**
**Cases**	**AD dementia**	**A+**	**A+T+**	**A+T+N+**	**MCI A+T+**	**SCD A+**
**Total (n)**	86	989	635	542	361	67
**Controls (n)**	43	455	256	271	151	50
**Cases (n)**	43	534	379	271	210	17
**AUC**	0.98	0.77	0.90	0.91	0.89	0.57
**95% CI**	0.97 to 1.00	0.72 to 0.81	0.88 to 0.93	0.88 to 0.93	0.85 to 0.93	0.40 to 0.74
**p value***	<0.0001	<0.0001	<0.0001	<0.0001	<0.0001	0.3834

*p value refers to the AUC of the ROC curve

Table S9. Comparison of the AUCs of the different ROC curves.

AUC comparison	Mean diff.	95% CI	p value (two-tailed)
**Hanley & McNeil test**			
ROC 1 vs. ROC 2	0.21	0.18 to 0.25	< 0.0001
ROC 1 vs. ROC 3	0.08	0.05 to 0.11	< 0.0001
ROC 1 vs. ROC 4	0.08	0.05 to 0.11	< 0.0001
ROC 1 vs. ROC 5	0.09	0.05 to 0.14	< 0.0001
ROC 1 vs. ROC 6	0.41	0.25 to 0.58	< 0.0001
ROC 2 vs. ROC 3	-0.13	-0.17 to -0.09	< 0.0001
ROC 2 vs. ROC 4	-0.13	-0.17 to -0.09	< 0.0001
ROC 2 vs. ROC 5	-0.12	-0.16 to -0.07	< 0.0001
ROC 2 vs. ROC 6	0.20	0.03 to 0.37	0.0191
ROC 3 vs. ROC 4	-0.00	-0.04 to 0.03	0.8373
ROC 3 vs. ROC 5	0.01	-0.03 to 0.06	0.5778
ROC 3 vs. ROC 6	0.33	0.16 to 0.50	0.0001
ROC 4 vs. ROC 5	0.02	-0.03 to 0.06	0.4768
ROC 4 vs. ROC 6	0.33	0.17 to 0.50	0.0001
ROC 5 vs. ROC 6	0.32	0.15 to 0.49	0.0003

Table S10. Comparison of log transformed z-scored plasma pTau181 levels between converters / non-converters' subjects with MCI in the modelling, testing and validation cohort.

Unpaired t test with Welch's correction	Modelling cohort	Testing cohort	Validation cohort*
p value (two-tailed)	<0.0001	<0.0001	<0.0001
Welch-corrected t	6.24	10.07	5.45
How big is the difference?			
Mean of MCI converters	0.39	0.43	0.40
Mean of MCI non-converters	-0.07	-0.44	-0.30
Difference between means (B - A)	-0.47	-0.87	-0.70
95% CI	-0.62 to -0.32	-1.05 to -0.70	-0.95 to -0.45
R squared (eta squared)	0.13	0.25	0.13
F test to compare variances			
F	1.45	1.03	1.18
p value	0.0043	0.8753	0.3740

***** Data only available in the GR@CE/DEGESCO cohort.

References

1. Folstein MF, Folstein SE, McHugh PR. “Mini-mental state”. A practical method for grading the cognitive state of patients for the clinician. J Psychiatr Res. 1975;12(3):189–98.

2. Blesa R, Pujol M, Aguilar M, Santacruz P, Bertran-Serra I, Hernández G, et al. Clinical validity of the “mini-mental state” for Spanish speaking communities. Neuropsychologia. 2001;39(11):1150–7.

3. del Ser Quijano T, Sánchez-Sánchez F, García de Yébenes MJ, Otero-Puime A, Zunzunegui M V, Muñoz DG. Spanish version of the 7 Minute screening neurocognitive battery. Normative data of an elderly population sample over 70. Neurologia. 2004;19(7):344–58.

4. Boada M, Tárraga L, Modinos G, López OL, Cummings JL. Neuropsychiatric Inventory-Nursing Home version (NPI-NH): Spanish validation. Neurologia. 2005;20(10):665–73.

5. Hachinski VC, Lassen NA, Marshall J. Multi-infarct dementia. A cause of mental deterioration in the elderly. Lancet. 1974;2(7874):207–10.

6. Blessed G, Tomlinson BE, Roth M. The association between quantitative measures of dementia and of senile change in the cerebral grey matter of elderly subjects. Br J Psychiatry. 1968;114(512):797–811.

7. Morris J. The Clinical Dementia Rating (CDR): current version and scoring rules. Neurology. 1993;43(11):2412–4.

8. Alegret M, Espinosa A, Valero S, Vinyes-Junqué G, Ruiz A, Hernández I, et al. Cut-off Scores of a Brief Neuropsychological Battery (NBACE) for Spanish Individual Adults Older than 44 Years Old. PLoS One. 2013;8(10):e76436.

9. Alegret M, Espinosa A, Vinyes-Junqué G, Valero S, Hernández I, Tárraga L, et al. Normative data of a brief neuropsychological battery for Spanish individuals older than 49. J Clin Exp Neuropsychol. 2012;34(2):209–19.

10. Jessen F, Amariglio RE, Boxtel M van, Breteler M, Ceccaldi M, Chételat G, et al. A conceptual framework for research on subjective cognitive decline in preclinical Alzheimer’s disease. Alzheimers Dement. 2014;10(6):844–52.

11. Espinosa A, Alegret M, Valero S, Vinyes-Junqué G, Hernández I, Mauleón A, et al. Longitudinal follow-up of 550 mild cognitive impairment patients: Evidence for large conversion to dementia rates and detection of major risk factors involved. J Alzheimers Dis. 2013;34(3):769–80.

12. Petersen RC. Mild cognitive impairment as a diagnostic entity. J Intern Med. 2004;256(3):183–94.

13. López O. Clasificación del deterioro cognitivo leve en un estudio poblacional. Rev Neurol. 2003;37:140–4.

14. Lopez OL, Kuller LH, Becker JT, Dulberg C, Sweet RA, Gach HM, et al. Incidence of Dementia in Mild Cognitive Impairment in the Cardiovascular Health Study Cognition Study. Arch Neurol. 2007;64(3):416–20.

15. de Rojas I, Moreno-Grau S, Tesi N, Grenier-Boley B, Andrade V, Jansen IE, et al. Common variants in Alzheimer’s disease and risk stratification by polygenic risk scores. Nat Commun. 2021;12(1):3417.

16. Diagnostic and Statistical Manual of Mental Disorders (DSM-5-TR) [Internet]. Ameriacan Psychiatric Association. 2023. Available from: https://www.psychiatry.org/psychiatrists/practice/dsm

17. McKhann GM, Knopman DS, Chertkow H, Hyman BT, Jack Jr CR, Kawas CH, et al. The diagnosis of dementia due to Alzheimer’s disease: recommendations from the National Institute on Aging-Alzheimer’s Association workgroups on diagnostic guidelines for Alzheimer’s disease. Alzheimers Dement. 2011;7(3):263–9.

18. Román GC, Tatemichi TK, Erkinjuntti T, Cummings JL, Masdeu JC, Garcia JH, et al. Vascular dementia: diagnostic criteria for research studies. Report of the NINDS-AIREN International Workshop. Neurology. 1993;43(2):250–60.

19. Neary D, Snowden JS, Gustafson L, Passant U, Stuss D, Black S, et al. Frontotemporal lobar degeneration: a consensus on clinical diagnostic criteria. Neurology. 1998;51(6):1546–54.

20. McKeith IG, Boeve BF, Dickson DW, Halliday G, Taylor J-P, Weintraub D, et al. Diagnosis and management of dementia with Lewy bodies: Fourth consensus report of the DLB Consortium. Neurology. 2017;89(1):88–100.

21. Pascual-Lucas M, Allué JA, Sarasa L, Fandos N, Castillo S, Terencio J, et al. Clinical performance of an antibody - free assay for plasma Aβ42 / Aβ40 to detect early alterations of Alzheimer ’ s disease in individuals with subjective cognitive decline. Alzheimers Res Ther. 2023;15(1):2.
